# Explainability and Transparency of Classifiers for Air-Handling Unit Faults Using Explainable Artificial Intelligence (XAI)

**DOI:** 10.3390/s22176338

**Published:** 2022-08-23

**Authors:** Molika Meas, Ram Machlev, Ahmet Kose, Aleksei Tepljakov, Lauri Loo, Yoash Levron, Eduard Petlenkov, Juri Belikov

**Affiliations:** 1 R8Technologies OÜ, 11415 Tallinn, Estonia; 2 Department of Software Science, Tallinn University of Technology, 12618 Tallinn, Estonia; 3 The Andrew and Erna Viterbi Faculty of Electrical & Computer Engineering, Technion—Israel Institute of Technology, Haifa 3200003, Israel; 4 Department of Computer Systems, Tallinn University of Technology, 12618 Tallinn, Estonia

**Keywords:** buildings, HVAC, fault detection and diagnosis, machine learning, explainable artificial intelligence, XAI, SHAP, XGBoost

## Abstract

In recent years, explainable artificial intelligence (XAI) techniques have been developed to improve the explainability, trust and transparency of machine learning models. This work presents a method that explains the outputs of an air-handling unit (AHU) faults classifier using a modified XAI technique, such that non-AI expert end-users who require justification for the diagnosis output can easily understand the reasoning behind the decision. The method operates as follows: First, an XGBoost algorithm is used to detect and classify potential faults in the heating and cooling coil valves, sensors, and the heat recovery of an air-handling unit. Second, an XAI-based SHAP technique is used to provide explanations, with a focus on the end-users, who are HVAC engineers. Then, relevant features are chosen based on user-selected feature sets and features with high attribution scores. Finally, a sliding window system is used to visualize the short history of these relevant features and provide explanations for the diagnosed faults in the observed time period. This study aimed to provide information not only about what occurs at the time of fault appearance, but also about how the fault occurred. Finally, the resulting explanations are evaluated by seven HVAC expert engineers. The proposed approach is validated using real data collected from a shopping mall.

## 1. Introduction

In recent years, we have witnessed an increasing interest in explainable AI (XAI) research in transportation, healthcare, legal, energy, finance, cybersecurity and other engineering domains [1,2,3,4,5,6,7]. With artificial intelligence dominating in major fields, it becomes imperative to create AI models that are transparent in the sense that the user is presented with an explanation of why the model generated a certain output or made a specific decision, all while preserving the high performance and accuracy of the model.

In building energy system applications, data-driven approaches have gained popularity as high-performing methods for the fault detection and diagnosis of HVAC systems [8]. The techniques include statistical-based approaches [9,10] and machine-learning-based approaches [11,12,13,14,15,16,17,18,19]. Table 1 provides a summary of fault diagnosis methods for air-handling units used in the literature, along with the fault classes and the numerical performance results. Machine learning approaches have received increasing interest for FDD problems due to their ability to capture non-linear relationships in multi-dimensional data, and they have outperformed traditional methods in the same task [8,20]. In Ref. [5], convolutional neural networks were applied to detect faults in the power system. XGBoost was used in the fault detection of the photovoltaic panels [21], and the result was significantly improved when an explainable method was added. In electric vehicle applications, deep learning was adopted for the fault diagnosis task [20], and it has been proven that diagnosing faults using the deep learning method shows better accuracy than the traditional fault diagnosis method. Another data-driven model was introduced in [22], which studied the intelligent fault detection system for microgrids. The authors of [23] developed a fault detection approach with an application in wind turbines, with a focus on data-driven techniques to overcome the problems of non-linearity, unknown disturbances, and noise in the wind turbine measurements. In building HVAC systems, data-driven methods have already become prevalent in the industry due to the ability to leverage large amounts of raw data [6]. While data-driven FDD models have ample potential when applied to complex HVAC systems, they may lack the ability to convince users to take actions towards energy efficiency. They have the ability to recognize patterns in the data and can make accurate predictions [8]. However, it is often the case that the models are trained to maximize their performance and accuracy over the training set. It becomes difficult to tell whether the high accuracy is due to over-fitting problems. The black-box nature of such models, combined with false-positive results, could potentially hinder users from trusting the system. Therefore, the improvement of model accuracy and the analysis of the model should work in parallel to increase reliability.

Several recent papers on fault detection and diagnosis for building energy systems have focused on explainability in order to gain user trust. XAI methods have been adopted to visualize the model output predictions of individual samples. Fault samples are classified using machine learning models, and XAI methods are used to explain individual samples by visualizing the contribution of each feature to the final output. This can help users to understand whether the prediction should be trusted. In work [6], the local interpretable model-agnostic explanations (LIME) framework was adopted to explain cases of incipient faults, sensor faults, and false-positive results from the diagnosis model for the chiller system, which is based on the XGBoost model. The general XAI-FDD workflow was validated using several real test cases. The proposed approach allowed a reduction in the manual fault-detection time, the analysis of the sources and origins of the problems, and the improvement of maintenance planning. The authors of [25] used the LIME method to explain the fault classification results of the support vector machine and neural network models developed for the diagnosis of heat-recycling systems. The method was able to explain the diagnosis of the component faults using examples of individual instances. In work [18], a new absolute gradient-weighted class activation mapping (Grad-Absolute-CAM) method was proposed to visualize the fault diagnosis criteria and provide fault-discriminative information for the explainability of the 1D-CNN model, applied to the detection of faults in chiller systems. The developed method was validated with an experimental dataset of an HVAC system, showing high diagnosis accuracy for seven chiller faults. The proposed method was able to successfully explain all of the fault criteria. In work [27], the Shapley additive explanation (SHAP) framework was adopted to explain four types of faults in the air-handling unit. The method, validated using real building data, explored the potential of adopting XAI methods in real-life building applications.

Although state-of-the-art explainable frameworks [6,18,25] have been applied to improve reliability in the fault detection domain, their concepts are still designed for AI researchers instead of non-AI experts [4]. Since the algorithm was developed for AI purposes, the concept may still be difficult to grasp by non-AI users. There remains a gap in the delivery of sufficient explanations, which impedes the adoption of XAI methods. State-of-the-art XAI techniques are designed for machine learning engineers across various domains. They are beneficial for machine learning developers in understanding whether there is any bias in the training data or whether the model learns to capture the correct feature relationships. The XAI outputs can provide insights into the black-box models, but they are still used to communicate with AI experts [4]. Excessively complicated explanations may cause end-users to use the system less. Therefore, outputs needs to be processed further before useful information can be extracted and delivered to target users. Another challenge in XAI is the creation of explanations that are trustworthy and accurate. A misleading explanation can cause users to misunderstand or lose confidence in using the system. If the machine learning model is developed using noisy data, the explanations generated from the model may also be of similar usefulness. Therefore, developing a machine learning model and understanding the reasoning behind the model should be addressed in parallel.

This paper presents a method to explain the decisions of an XGBoost-based classifier using SHAP as the interpreter. The method could benefit end-users by requiring justification and reasoning behind the fault predictions. For interpretability in the context of HVAC fault detection with time-series data, we visualized a sliding window to provide explanations for the observed faults. The aim was to provide insights into not only what happens in a single time step, but also to understand what has happened prior to the observation that leads to the fault occurring. To keep the explanation information focused, we only showed the user-defined sets of features corresponding to the observed fault and the features with high contribution scores. The experiment was conducted on the real-world data of an air-handling unit containing normal and faulty samples. The explanations were applied to samples of each fault type and then assessed by HVAC experts. To summarize, the main contributions of this work to the field of explainable fault detection and diagnosis methods for building energy systems are outlined as follows:

1. A method to explain the fault diagnosis output of an XGBoost-based model using Shapley values for HVAC expert users. A sliding window system is used to visualize the short history of the relevant features and provide explanations for the diagnosed faults in the observed time period. This allows users to understand not only what happens in each individual time step but also to monitor the progress history of the fault. The fault detection and diagnosis pipeline is conducted using real-world data obtained from the air-handling unit of a commercial building.
2. A method to incorporate human users into the decision-making process by allowing the selection of relevant features to be explained for each fault type. The method explains the features with high Shapley values and features corresponding to each fault type. This can provide a practical value by keeping the explained features relevant.
3. An analysis for the XAI explanations of each fault type in the AHU dataset by using domain expert evaluation to obtain feedback on the generated explanations. This helps us understand the effects of the explanations on the users’ decision-making process, and how well the users can understand the explanations.

## 2. Technical Background: XGBoost and SHAP

### 2.1. Extreme Gradient Boosting

XGBoost is an ensemble model based on gradient tree boosting that works by integrating several basic classifiers together, usually decision tree models, to form a more robust model [28]. The model learns through an additive manner or a cumulative learning process. First, the starting tree is fitted with the entire training dataset. Then, the learning result of the tree is passed to the next tree to update the weights and the process is repeated. The final result is obtained by accumulating the results from all the trees. The prediction function in step *t* is denoted as:

(1) $$f_{i}^{\left(t\right)}=\sum_{k=1}^{t}f_{k}\left(x_{i}\right)=f_{i}^{\left(t-1\right)}+f_{i}\left(x_{i}\right),$$

where $f_{i}\left(x_{i}\right)$ is the tree model at step *t*, and $f_{i}^{\left(t\right)}$ and $f_{i}^{\left(t-1\right)}$ are the predicted values in steps *t* and t−1, respectively. To learn the sets of functions, XGBoost seeks to minimize the following objective:

(2) $$L\left(\phi \right)=\sum_{i=1}^{n}l\left({\hat{y}}_{i},y_{i}\right)+\sum_{k=1}^{m}\Omega \left(f_{k}\right),$$

where

(3) $$\Omega \left(f\right)=\gamma T+\frac{1}{2}\lambda {∥w∥}^{2}.$$

Here, *n* is the number of training samples, $l({\hat{y}}_{i},y_{i})$ is the loss function, $\Omega (f_{k})$ is the regularization term on the *k*th decision tree, *w* is the score from leaf nodes, λ is the hyperparameter of the regularization term, and γ is the minimum loss that the leaf node needs to make further splits. The regularization term Ω penalizes the complexity of the model and smooths the final learned weight to avoid over-fitting. Without the regularization term, the objective function falls back to traditional gradient tree boosting [28].

For a faster calculation of the objective function, XGBoost uses the second-order Taylor’s expression in the loss function

(4) $$L^{\left(t\right)}≃\sum_{i=1}^{n}\left[l\left(y_{i},{\hat{y}}^{\left(t-1\right)}\right)+g_{i}f_{t}\left(x_{i}\right)+\frac{1}{2}h_{i}f_{t}^{2}\left(x_{i}\right)\right]+\Omega \left(f_{t}\right).$$

### 2.2. Shapley Additive Explanation

SHAP is a game-theory-based approach to explain the individual predictions produced by machine learning models [29]. SHAP was applied in various fields such as medicine, energy systems, and the fault detection domain [30,31,32]. It can be conveniently applied to any black-box machine learning model and to different types of data, e.g., tabular, image, or text data, since it is model-agnostic. It is used to show the contributions of the input features using the computed Shapley values, where each feature works together in a coalition. The Shapley value is calculated for each feature in the input samples that needs to be explained. Based on the aggregated Shapley values, it can also provide global interpretations of the black-box models. SHAP describes three desirable properties, namely local accuracy, missingness, and consistency, as follows [29]:

1. **Local accuracy**: When approximating the original model *f* for an input *x*, local accuracy is the ability of the explanatory model to represent the output of the simplified model $f^{\prime }$ for the simplified input $x^{\prime }$:
  (5) $$f\left(x\right)=g\left(x^{\prime }\right)=\phi_{0}+\sum_{i=1}^{M}\phi_{i}x_{i}^{\prime }.$$
2. **Missingness**: Missingness requires that the features that are missing from the input to have zero impact on the model output:
  (6) $$x_{i}^{\prime }=0\Rightarrow \phi_{i}=0.$$
3. **Consistency**: Let $f_{x}\left(z^{\prime }\right)=f\left(h_{x}\left(z^{\prime }\right)\right)$ and $z^{\prime }\backslash i$ denote setting $z_{i}^{\prime }=0$. For any two models *f* and $f^{\prime }$, if
  (7) $$f_{x}^{\prime }\left(z^{\prime }\right)-f_{x}^{\prime }\left(z^{\prime }\backslash i\right)\geq f_{x}\left(z^{\prime }\right)-f_{x}\left(z^{\prime }\backslash i\right)$$
  for all inputs $z^{\prime }\in {\left\{0,1\right\}}^{M}$ with *M* being the number of simplified features, then $\phi_{i}\left(f^{\prime },x\right)\geq \phi_{i}\left(f,x\right)$. The consistency property states that if a model changes, so that the marginal contribution of a feature increases or stays the same, regardless of the other inputs, the attribution of that input feature should also increase or stay the same.

The author of SHAP also proposed TreeSHAP as a fast and model-specific method to calculate SHAP values from tree models such as decision trees and other ensemble tree models. Given a data instance *X* with features $x_{1},x_{2},\ldots ,x_{n}$, and a classifier model *f*, TreeSHAP receives *f* as the input to obtain the explanatory object. Then, such an object takes *X* as the input and generates the SHAP values. The process is depicted in Algorithm 1.
**Algorithm 1.** Calculating SHAP values**Input:***X*: instance for which the explanations are generated  *f*: classification model for fault diagnosis**Output:**shapVal: SHAP values for all features  1: explainer←shap.TreeExplainer(f)  2: shapValue←explainer.shapvalues(X)


## 3. Methodology

This section describes the method for communicating the explanation to the end-users and the human evaluation of the explanation. The major steps in the process include the following: data collection and preprocessing; the implementation of XGBoost; domain requirements for the explanations; SHAP explanations; and the expert evaluation of the explanations. First, an FDD model based on the XGBoost classifier is implemented. The FDD process is completed in a single step, with the diagnosis model classifying the five types of faults and the normal operation state. We conducted a case study using real data collected from a commercial building (a shopping mall). Then, the classification result is summarized using various performance metrics. Next, the samples of five different types of faults in the AHU are selected to provide explanations. The SHAP method is integrated as the explanation algorithm. The output of SHAP is visualized for each fault type based on sliding window observation using the most relevant features. Finally, fault samples are randomly chosen to generate SHAP explanations, using different visualization techniques. The generated explanations are assessed by HVAC engineers, who give feedback and assess the effect of the explanations on their decision-making process.

**Figure 1:**
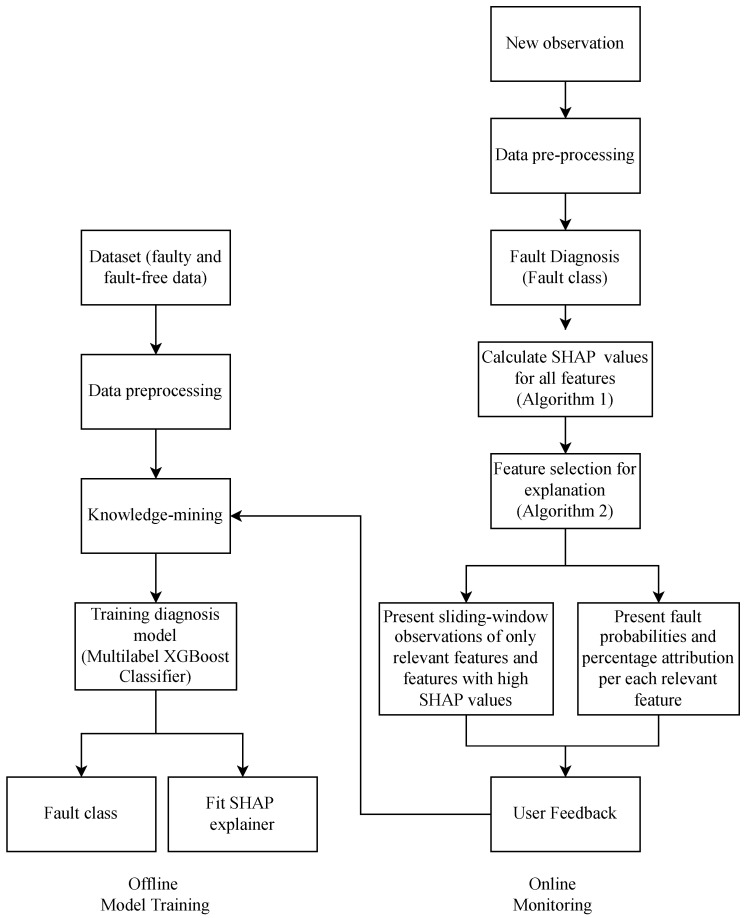
Proposed explainable fault detection and diagnosis pipeline. The left-hand side represents the offline model training stage, while the right-hand side corresponds to the online fault monitoring stage.

Figure 1 outlines the proposed methodology, which can be summarized as follows:

1. Offline model training stage:
  (a) Data are collected for faulty and fault-free operations and labeled according to fault type. Samples that do not belong to any fault class are labeled as normal. Data are preprocessed by removing records with null or non-existing values. Samples collected during the off-state and during the first hour of operation are also removed.
  (b) Prior knowledge related to all fault types is gathered. This includes the mapping of feature sets that correspond to each type of fault. The feature sets can be input by end-users, who decide which relevant features they want to see for each type of fault; otherwise, the default feature sets are chosen.
  (c) An XGBoostClassifier model is implemented for the FDD problem. The model is a multi-class multi-label classification model, which is used to classify which fault class(es) each sample belongs to. One sample can belong to multiple fault classes.
  (d) The SHAP method is used to generate explanations for the fault diagnosis model. A Tree SHAP explanatory object is fit using the developed model to be able to generate explanations during the online monitoring stage.
2. Online fault monitoring stage:
  (a) Real-time measurements are obtained from the system. The new observation is preprocessed and input into the trained XGBoost model to distinguish between the fault classes and the normal class.
  (b) If the sample represents a faulty operation, the interpreter module is triggered to generate the explanation. If no fault is detected, the following steps are skipped.
  (c) Using the fitted SHAP explanatory object from the previous offline training stage, SHAP values are generated for the observed faulty samples and the samples from a number of time steps prior to observation, to provide a short history of fault occurrence.
  (d) A visualization is created for relevant features using a sliding window graph.
  (e) A user gives feedback on the explained feature choices, which is used to update the sets of relevant features.

### 3.1. Description of the System

In this paper, we consider the data obtained from a shopping mall that was renovated over a decade ago. The facility has three floors that are mostly heated by a group of air-handling units. The building is heated with district heating while the cooling is provided by two chillers.

Almost every large commercial building has a building management system (BMS), which contains thousands of data points that are presented through a user interface in real time. A BMS is usually devoted to information flow and communication with the HVAC equipment. In addition to monitoring, it also provides custom reactive alarms to notify the operators at different levels. Data acquisition in the facilities is accomplished through the dedicated BMS. Data reading and writing is conducted through the API connection, supported by the BMS. Remote connection via APIs varies depending on the deployed software, each of which requires custom solutions for reliable data communication. Finally, the data transmission is secured through encrypted virtual private network tunnels. Data through the BMS are read every 15 min, and samples of an air-handling unit were collected during the period from 1 February 2020 to 31 March 2021. This includes measurements obtained from an air-handling unit during the winter, spring, summer, and autumn seasons in Estonia. In commercial buildings, AHUs usually follow an operating schedule that commands that the system be switched on during an occupied period and off during an unoccupied period. Before the analysis, the data were filtered to exclude extreme outliers and samples taken during non-operating periods. The data were further processed and the faults were labeled by a dedicated HVAC engineer.

The dataset includes 10 input features, as shown in Table 2. This contains the samples of an air-handling unit under normal operating conditions and five types of faults, as listed in Table 3. The fault cases are taken from real scenarios and operating conditions.

### 3.2. Air-Handling Unit

**Figure 2:**
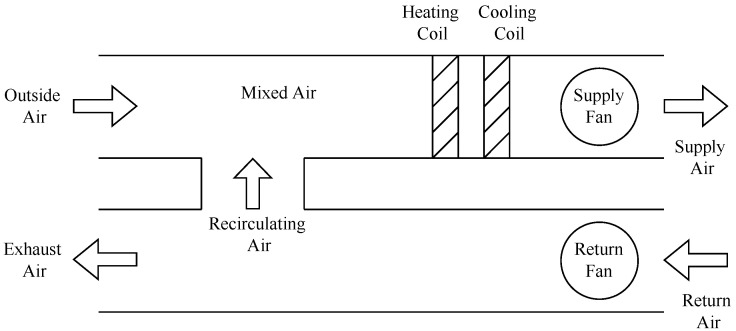
Schematic diagram of an air-handling unit.

Figure 2 shows a schematic diagram of one of the variable-air-volume (VAV) air-handling units utilized in this study. The AHU consists of fans, dampers, cooling and heating coils, sensors, controllers, and heat recovery units. The supply fan draws fresh air from outside, and the air is passed through a fan filter to filter dust or objects that could cause damage or inefficiency inside the system. Then, the heating coil and cooling coil valves are either opened or closed in order to maintain the desired setpoint temperature of the supply air. For example, when the supply air temperature is excessively low, the heating coil valve opens to allow hot water to flow through the heating coil and heat up the supply air until the setpoint is reached. The conditioned supply air is then distributed to different zones or rooms in the building. Several factors can affect indoor air quality. If the room is occupied, heat might radiate from the body and cause a high room temperature. This occupation may also affect the CO_2_ and humidity levels, and the room may become uncomfortable again. Therefore, the air in the room needs to be circulated back while letting fresh air in. The return fan draws the air from the zone back to the unit. Then, the return air is either recirculated and mixed with the fresh air in the mixing box, or is drawn away as exhaust air [10,11,33].

In order to maintain energy efficiency, the heat recovery system is utilized to recycle the return air by mixing it with the fresh intake air in the mixing box. That way, some energy can be recovered without having to activate the heating system every time heating is required, since using the heating system is more energy-demanding than using heat recovery. In the ventilation unit used for this study, the heat recovery system used is a rotary system. The efficiency of the heat recovery process is an indicator of the amount of energy that it manages to recycle [25,33].

### 3.3. Data Preprocessing

The dataset includes 13 input features, as shown in Table 2. These include samples of an air-handling unit under normal operating conditions and five types of faults, as listed in Table 3. The fault cases are taken from real scenarios and operating conditions.

The input data are split into 66% and 34% for the training and test sets, respectively. Randomly stratified sampling is applied in the data partitioning process to maintain the balance of fault classes for both sets.

Table 3 shows that the number of samples of normal operation (majority class) exceeds that of faulty cases (minority class), with an extreme imbalance. Having such imbalanced classes for classification problems can lead to biased predictions towards the majority class. This problem is tackled with random under-sampling techniques to transform the class distribution in the training set and eliminate the extreme data imbalance.

### 3.4. Description of Faults under Observation

Five faults representing failures in the sensor, heat recovery, heating coil, and the cooling coil valve of the AHU are described. The faults are introduced when the system is supposed to be operating under steady-state conditions and the symptoms of dominant features that correspond to the fault occur. All faults described here are representations of actual fault scenarios from an air-handling unit of a commercial building.

Fault 1 is a malfunction of the fan pressure sensor. The fan pressure sensor measurement is used to calculate the control value for the supply or return fan speed. The fault causes the control signal for the supply fan speed to fall in an undesirable range. Thus, the important features in this fault include the fan pressure (ASFPE) and the fan speed (ASFS).

Fault 2 is the failure of the heat recovery. During normal operation, the heat recovery should operate at 70% efficiency or above. The heat recovery is utilized before the heating coil is used. The important features in determining this fault include heat recovery speed (AHRS), return air temperature (ARAT), ambient temperature (AAT), and supply air temperature (ASAT).

Fault 3 is the heating coil valve leakage. The fault indicates that the heating coil valve is not closing completely when there is a command to close it. Regardless of the fact that the valve should be closed, the hot water flows through the coil and heats up the supply air. This results in an extra heating cost and may even lead to extra cooling costs and the undesired temperature of the supply air. The leak can be detected by checking the temperature sensors in the supply air channel, or comparing the work of the heat recovery and cooling coil with other ventilation machines or this machine’s typical actions. The important features of this fault include the heating coil valve opening (AHCVO), supply air temperature (ASAT), and supply air temperature after heat recovery (AHRST).

Fault 4 is the stuck cooling coil valve. The fault indicates that the cooling valve is stuck at a lower value and the ventilation unit is not fully utilizing the cooling capacity. The important features in this fault include the cooling coil valve opening (ACCVO), supply air temperature (ASAT), and calculated supply air temperature setpoint (ASATCSP).

Fault 5 is the closed cooling coil. This fault implies that the ventilation unit controller is not sending a command to fully utilize the cooling capacity. This might indicate a problem with the controller. The important variables in this fault type include the cooling coil valve opening (ACCVO), supply air temperature (ASAT), and the supply air temperature setpoint (ASATCSP).

Based on the complete feature set, which includes original features and extracted features, it is possible to generate a feature map, as shown in Table 4, which maps the features to their corresponding fault types using domain knowledge. This will serve as crucial information in a later step when communicating the fault prediction to end-users.

### 3.5. XGBoost and Hyperparameter Tuning

In this study, the model aims to predict whether the AHU is operating in a normal or faulty condition at specific timestamps, and to determine which fault type(s) are present. For training the fault diagnosis model, the problem is formulated as a multi-label classification problem, where the labels are binary vectors (value of 0 or 1 for each of the five fault classes, plus the normal class), and more than one fault type(s) can be present simultaneously. The training set is used to train the XGBoost model. The hyperparameter is tuned as follows: the number of estimators used is 10. In order to reduce the over-fitting problem, the column subsample ratio is set to 0.9 and the alpha regularization term is set to 0.005.

### 3.6. Explaining the Fault Predictions

Our challenge is to design explanations and deliver relevant information that can help target users to identify faults. The target users in our case are the HVAC engineers, as opposed to the machine learning engineers. We implemented a fault diagnosis model to classify the faults in the AHU using a supervised learning approach. If a fault exists, the SHAP explanatory object is triggered to provide an explanation using the sliding window method. Here, we use Tree SHAP to generate the SHAP values, which describe the attribution score for each feature in predicting the fault classes.

The overall fault detection, diagnosis, and monitoring steps are shown in Figure 1. We focused on the XAI layer, which is applied to visualize the fault decision in the monitoring stage after the fault diagnosis model outputs the fault class. The explanations are given for any selected individual fault instance. First, the SHAP values are calculated for each of the features in the fault instance, as specified in Algorithm 1. Next, we performed feature selection to narrow the features down to only the most relevant, which will be visualized in the explanatory graph in the next step. The feature selection process is described in Algorithm 2. The authors of [31] described the process of selecting features used to explain an autoencoder-based anomaly detection model. In our work, the feature selection for explanations is based on:

- Features that have SHAP values higher than mean SHAP values.
- Features that are pre-selected by users or features that are mapped to each corresponding fault type using prior knowledge.

**Algorithm 2.** Feature selection for providing explanations**Input:** userSelectedFeatures: user-selected features,  shapValue: SHAP values for all features (see Algorithm 1),  features: list of all features**Output:** relevantFeatures: relevant feature list  1:
$\mathrm{avgShapVal}\leftarrow \bar{\mathrm{shapValue}}$  2:
shapFeatures←{}  3:
**for** 
feature∈features 
**do**  4:
   **if** shapValue[feature]>avgShapVal
**then**  5:
    shapFeatures←feature  6:
   **end if**  7:
**end for**  8:
relevantFeatures←shapFeatures∪userSelectedFeatures  9:
**return**
relevantFeatures

After we obtained the set of relevant features and their corresponding SHAP values, we presented the results to the end-users using a sliding window visualization technique. We plotted the actual values of the obtained relevant features into the sliding window graph, which allows users to view the equipment conditions over the observed time window.

For the selected fault, we present its class, the corresponding fault probability, and how much each relevant feature contributes to the presence of the fault. Since the obtained raw SHAP values are not easily comprehensible, the values are rescaled into percentage contributions using a logistic transformation and the percentage attributions are displayed instead.

### 3.7. Domain Requirements

We conducted a survey with seven HVAC engineers (E1–E6) who are actively working with HVAC systems. We provided a survey form which contains a list of criteria that fault diagnosis explanations may contain. The complete list can be found in the Appendix A. The participants were asked to rate how important these criteria are, and to add more criteria that they think are necessary. First, we will cover some of the feedback from the participants.

E1 and E5 are interested in viewing the fault impact in terms of the cost. More specifically, both participants mention that the cost factor is convincing when it comes to fault diagnosis. Knowing how many financial consequences will be created from not fixing the fault will motivate the users to take action. Other types of fault impact analysis, such as indoor climate impact, could also be convincing factors. However, this can be regarded as a topic for future work, since it is out of the scope of this thesis. E2 would like the option to choose more variables to visualize the explanations, on top of visualizing only the most important variables. E5 would like to view the measured data in a graph with the relevant variables within one hour prior to the fault and one hour after the fault occurs. The idea is to observe when the fault probability changes and to visualize in what condition the fault is detected. E6 feels that it is important to know how often the fault has occurred previously in order to identify whether the problem is instead a result of other problems. E7 would like to see the fault-free sample to compare it with the faulty sample, in order to understand the expected value.

We compiled the following requirements from the feedback and the rating of each criteria:

**R1**: Option to choose variables: E2, E3, E5, and E6 would like to have the option to choose variables. E2 thinks that this option becomes less important if the result shows the most relevant variables by default. Important
**R2**: Visualizing the short history of faults: E1, E5, E6, and E7 think that viewing the short history of faults is important. E2 thinks that this option is different from one fault type to another. In real life, some faults occur so suddenly that in this case, the short history of the fault is useful. Important
**R3**: Visualizing only relevant variables: E1, E2, E3, E4, and E7 would like to view only the most important features that impact the fault likelihood. In addition to **R1**, they only want to view the variables that influence the fault, and to have the option to select more variables. Critical
**R4**: Visualizing each feature’s attribution to the fault: E2, E4, and E5 think it is good to view how much each feature affects the fault likelihood in terms of probability. In addition, E2 also specifies that the probability of the diagnosed fault is also a convincing factor for users. For example, if the probability is 1.00, then it is more convincing than when the probability is only 0.97. E3 and E4 do not think that this is very important. E5 would like to observe the change in probability when the fault occurs. Optional

## 4. Results and Analysis

In this section, we discuss the numerical results obtained from the trained model.

### 4.1. Performance Results

The performance is evaluated using the test set for the trained models—logistic regression, random forest, and XGBoost. The accuracy, precision, recall, sensitivity, specificity, and F1 scores are displayed in Table 5. The XGBoost method achieves the highest overall performance for most fault types.

**Figure 3:**
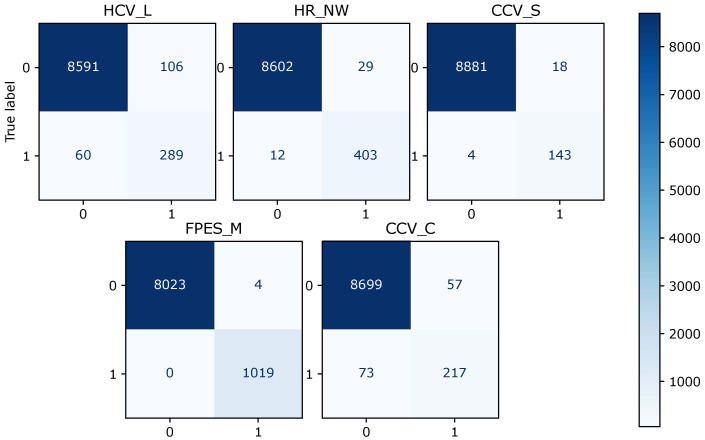
Diagram of the confusion matrix of different fault classes for the logistic regression model, for five fault types. The vertical axes represent the actual labels, and the horizontal axes represent the predicted labels for the corresponding fault classes. The total number of correctly predicted samples is the sum of all actual samples labeled as true and classified as true, and the actual samples labeled as false and classified as false.

**Figure 4:**
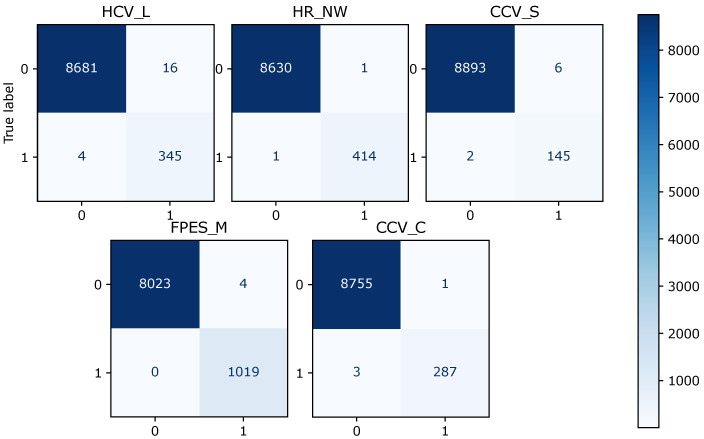
Diagram of the confusion matrix of different fault classes for the random forest model and for five fault types.

**Figure 5:**
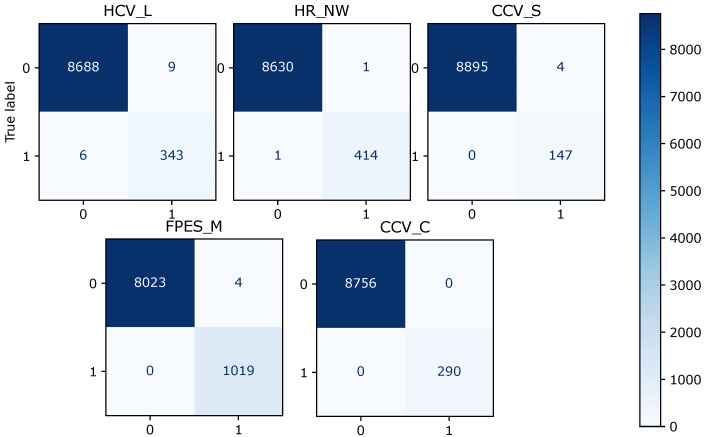
Diagram of the confusion matrix of different fault classes for the XGBoost model and for five fault types.

Table 5 shows that the overall F1 score for logistic regression is 0.88, random forest is 0.994, and XGBoost is 0.997. Logistic regression scores 0.85 in terms of the overall precision score, while random forest and XGBoost score 0.996 and 0.997, respectively. Logistic regression makes the prediction with an overall recall score of 0.90, random forest of 0.994, and XGBoost of 0.997. The confusion matrix for the model prediction of each fault type is shown in Figure 3, Figure 4 and Figure 5. For each matrix, the vertical axes represent the true labels, and the horizontal axes represent the predicted labels for the corresponding fault classes. It can be observed that the number of misclassifications for the XGBoost model is 25, that for random forest is 38, and that for the logistic regression model is 363.

### 4.2. Comparative Study

As shown in the confusion matrix and the performance matrix, XGBoost outperformed both of the baseline models. Random forest performance is also comparable to the XGBoost, with minimal differences in the score. However, in fault detection and diagnosis tasks, it is crucial to minimize the number of false positives by as much as possible. We chose XGBoost as our method of fault diagnosis in this study because of its high performance. In this section, we will study the model interpretability using SHAP. The interpretations of the baseline model, random forest, and XGBoost will also be analyzed and compared.

**Figure 6:**
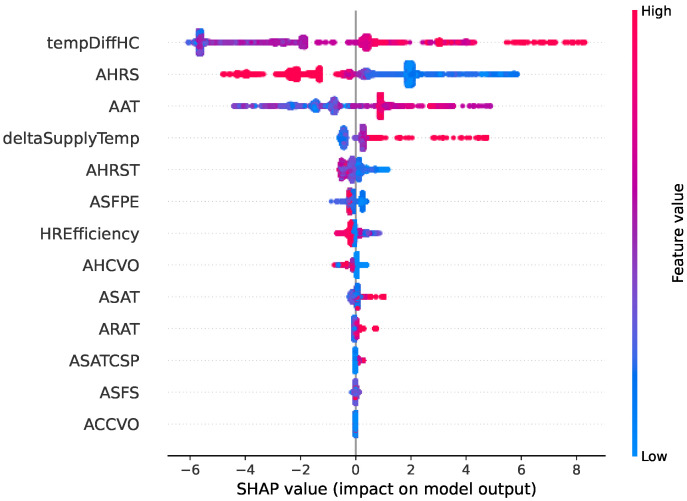
Summary of the effects of all the features on the XGBoost model prediction of the fault type “Heating coil valve leak”. The SHAP values show the impact of each feature on the model output. The color represents the feature value (red shows a higher impact while blue shows a lower impact). For example, we can observe that the higher the deltaSupplyTemp (difference between supply air temperature and its setpoint), the more likely it is to indicate a heating coil valve leak.

**Figure 7:**
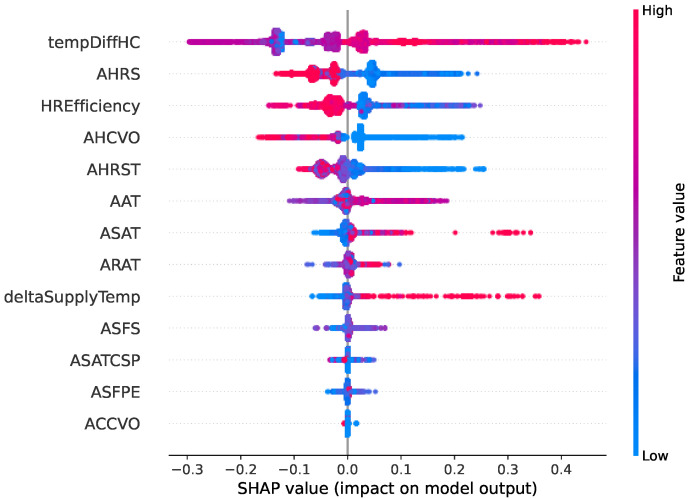
Summary of the effects of all the features on the random forest model prediction of the fault type “Heating coil valve leak”.

**Figure 8:**
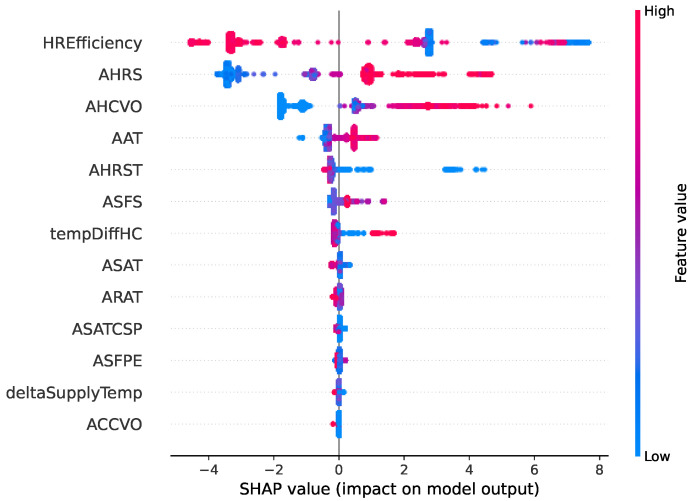
Summary of the effects of all the features on the XGBoost model prediction of the fault type “Heat recovery not working”.

**Figure 9:**
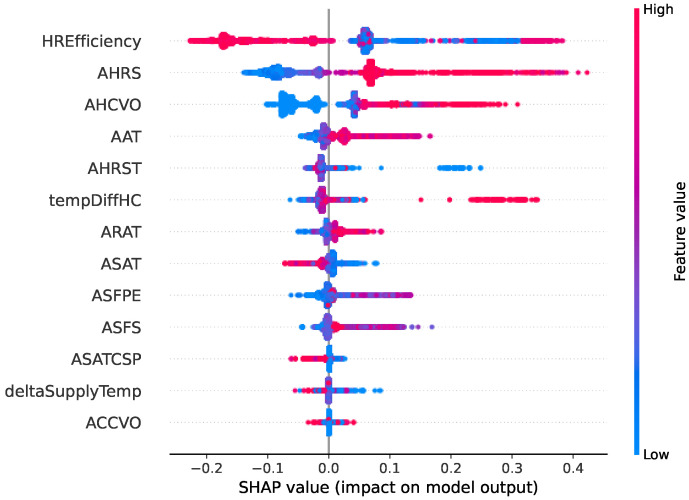
Summary of the effects of all the features on the random forest model prediction of the fault type “Heat recovery not working”.

Two types of faults are selected in this comparative study. Figure 6, Figure 7, Figure 8 and Figure 9 show SHAP summary plots for XGBoost and random forest models in predicting the two types of fault classes. The features are ranked by their importance in the model prediction. The *x* axis indicates the SHAP value where a positive SHAP value means a higher contribution to the fault and a negative value means a negative impact on the fault. For SHAP, the summary plot is made up of SHAP values from each individual sample. Therefore, this interpretation also represents the local interpretation. The red color indicates the high value of the variable. The red-colored dots most concentrated on the positive side of the SHAP value axis denote the higher values of the corresponding features, having a positive influence on the fault class. The opposite applies to the blue color. If the features are represented by blue dots concentrated on the positive side of the SHAP value axis, this indicates that the low values of these features contradict the fault instead.

Figure 6 and Figure 7 show the SHAP summary plot of the model prediction of the heating coil valve leakage fault type from the XGBoost model and random forest model, respectively. The plot shows that the two most important variables from both models are the same: tempDiffHC (temperature difference before and after the heating coil) and AHRS (heat recovery speed). In the XGBoost SHAP plot, the deltaSupplyTemp (difference between the supply air temperature and its setpoint) is among the top five features. Based on the plot, the greater the difference is, the more likely a heating coil valve leak is. The random forest explanation also indicates the same. The random forest gives more importance to heat recovery efficiency (HREfficiency). From the domain knowledge, the heat recovery status also gives some indication of the fault heating coil valve leakage since heat recovery should be working at full speed to recycle the heat before the heating system in the heating coil should be activated. The variable AHRS (heat recovery speed), as the second most important variable, should also be somewhat correlated with the HREfficiency.

The SHAP summary plot of the XGBoost model for predicting the fault “Heat recovery not working” is shown in Figure 8, and the random forest model SHAP summary plot for the same fault class is shown in Figure 9. Based on the figures, the top five most important features are the same between the two models. This includes the HREfficiency (heat recovery efficiency), AHRS (heat recovery speed), AHCVO (heat recovery valve opening), AAT (ambient temperature), and AHRST (supply air temperature after heat recovery). There are very slight differences in the pattern, but the overall feature effects share many similarities. For both of the models, the most important feature is HREfficiency. Domain knowledge confirms that HREfficiency is an important indicator of the fault malfunction of heat recovery. The rest of the features in the top five list fit into this fault description. In both summary plots, high AHRS and low HREfficiency are signs that there is a chance of malfunction in the heat recovery. For example, the high heat recovery fan speed suggests that it is rotating, but the low efficiency indicates that it fails to recycle any heat. The plots also imply that the high HREfficiency has a negative influence on the fault. This correlates with domain knowledge, since higher efficiency means that the fan is working properly.

This comparative analysis provides us with insights into what is taken into account by the model in predicting the fault classes. For data with very high dimensions, it can give useful information regarding how the change in value of the most important features can affect the model output. We observe from our two fault examples that the generated global explanations for both models share some similar patterns and feature importance. In the first fault case, three out of the five most important features are the same between both models. Furthermore, in the second fault case, five out of the five most important features are the same. Nonetheless, it is inconclusive to evaluate the models based on XAI explanations. One of the reasons is that there is a possibility that the generated explanations might not be fully representative of the actual model. Another reason is that there can be other underlying problems, such as inaccuracies in the dataset, over-fitting, or inadequacy in the hyperparameter tuning.

### 4.3. Explaining Faults

In this section, SHAP is used as the explainable method. Explanations are provided for the XGBoost model output of randomly chosen samples from each type of fault in the dataset. The visualization design is based on the domain requirements collected from the previous sections. The figures below visualize the explanations in a sliding window format. Instead of plotting the SHAP values, actual values are provided. To show the contributions of each feature to the fault, the SHAP value for each relevant feature is converted to a percentage and is added in the annotation. Fault probability is also provided for the instances that the user wants explanations for. The areas in which a fault is present are highlighted with a light red background to allow for an understanding of where the fault begins.

### 4.4. Case Studies

#### 4.4.1. Case 1: Fan Pressure Sensor Malfunction

**Figure 10:**
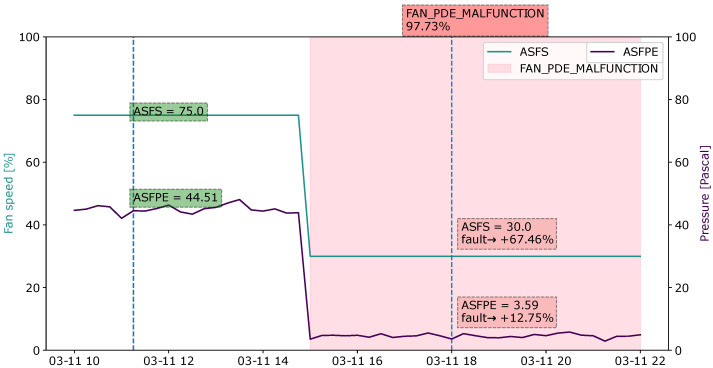
Visualization of explanations using a sliding window for the fault type “Fan pressure sensor malfunction” during an observation period from 10:00 to 22:00. Tags labeled in green represent a healthy state, whereas those highlighted in red represent the presence of the fault. The sample evaluated at 18:00 shows an overall fault probability of 97.73% with two main contributing features, ASFS and ASFPE.

Figure 10 shows the explanation for the “Fan pressure sensor malfunction” fault of the observed sample, which occurred at 18:00 on November 2003. To understand the progress history of the fault, the measurement values for a number of time steps, starting from 10:00 on 3 November, are shown. In this observed instance, the variables ASFS (supply fan speed) and ASFPE (supply fan pressure) have a positive impact on the fault prediction. The supply fan speed is 30% and the supply fan pressure is at 3.59 Pa during the hour of operation. The fault starts multiple time steps prior to the observed sample. Before the fault occurs, the fan and fan pressure measurements, which are 75% and 44.51 Pa, respectively, show normal operation up until the values of both variables suddenly drop to very low values, which indicate that the fan is barely operating. The low value of the ASFS contributes 68.26% to the fault probability, and the ASFPE contributes 12.9%. Based on the domain knowledge, the fan pressure and fan speed are two correlating features where the pressure measurement is a variable used for calculating the air volume. Therefore, the malfunction of the pressure sensor may also cause failure in the air volume control.

#### 4.4.2. Case 2: Heat Recovery Not Working

**Figure 11:**
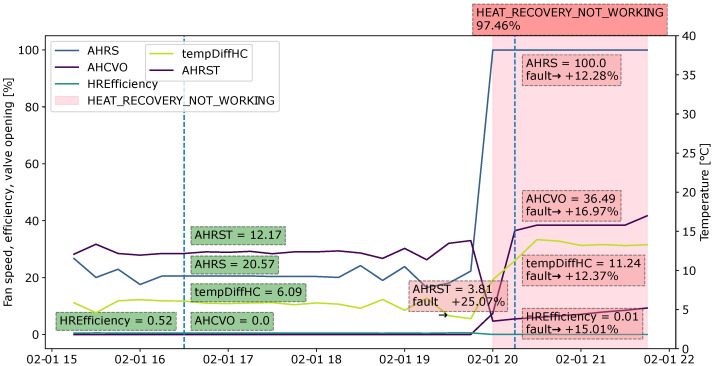
Explanation visualized as a sliding window for the fault type “Heat recovery not working” during an observation period from 15:00 to 22:00.

Figure 11 shows samples for the diagnosis of the heat recovery fault. The fault becomes very likely when the AHRS (heat recovery fan speed) is 100% and the HREfficiency (heat recovery efficiency) is only 0.01%, meaning that it fails to recover heat from the return air to heat up the supply air. When the air is not sufficiently heated by the heat recovery unit, the heating coil valve opens to produce the heat. The AHRST (air temperature after heat recovery) is very low at that point, although the heat recovery is working at full speed. The heat recovery efficiency contributes approximately 16% to the fault, the heat recovery speed contributes 12%, the air temperature after heat recovery contributes 26% and the temperature difference before and after the heating coil influences 13%.

#### 4.4.3. Case 3: Heating Coil Valve Leakage

**Figure 12:**
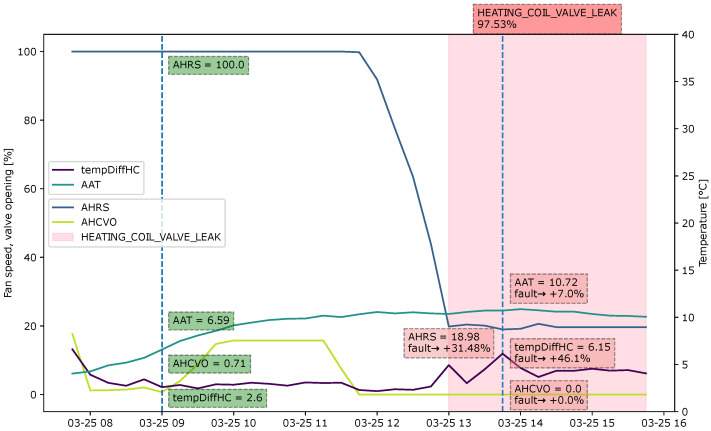
Explanation visualized as a sliding window for the fault type “Heating coil valve leak” during an observation period from 08:00 to 16:00.

Figure 12 represents the explanation for the fault “Heating coil valve leakage”. The heating coil opens for some time, from 09:00 to 11:45, to heat up the supply air before closing again. However, the temperature difference before and after the heating coil (tempDiffHC) increases, although the heating coil valve is 0. The heat recovery rotation is not utilized at 100% when the fault occurs. This could also indicate that the supply air is sufficiently heated and does not require much heat to be recycled. The low heat recovery rotation speed adds 27% to the fault. The high-temperature difference before and after the heating coil adds another 50%. Through the domain knowledge, this information is enough to determine the fault.

#### 4.4.4. Case 4: Cooling Coil Valve Stuck

**Figure 13:**
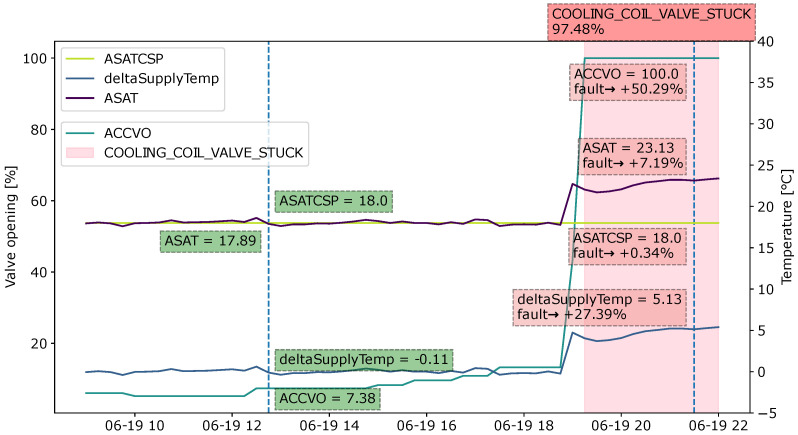
Explanation visualized as a sliding window for the fault type “Cooling coil valve stuck” during an observation period from 10:00 to 22:00.

Figure 13 shows the explanation for the “cooling coil valve stuck” fault. When the supply air temperature becomes higher than the setpoint at 19:00, the cooling coil valve opens in order to cool down the supply air. The cooling coil valve control signal shows 100%. However, the supply air temperature stays the same. Furthermore, by the time of observation at 21:30, the supply air temperature is 5.13 °C higher than the setpoint. The high temperature of the supply air adds 7.57% to the fault. The difference between the supply air temperature and setpoint temperature (deltaSupplyTemp) contributes 29%, and because the cooling coil valve is 100%, the fault probability increases by a further 53.64%. In contrast to the non-faulty sample shown at 12:45, the deltaSupplyTemp is only 0.11, which means that the supply air temperature achieves the setpoint.

#### 4.4.5. Case 5: Cooling Coil Valve Closed

**Figure 14:**
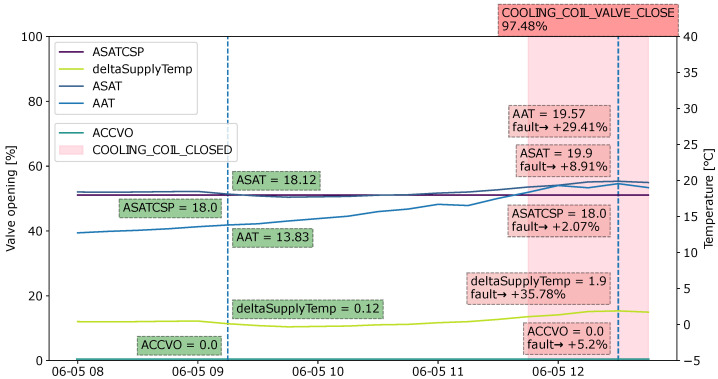
Explanation visualized as a sliding window for the fault type “Cooling coil valve closed” during an observation period from 08:00 to 12:45.

The example of a “cooling coil valve closed” fault is given in Figure 14. From the graph, the temperature difference between the supply air and setpoint (deltaSupplyTemp) increases as the ambient temperature (AAT) increases. The supply air temperature (ASAT) becomes higher. The system should send the command to open the cooling coil valve and regulate the supply air temperature to the setpoint level. However, the valve remains closed for some time steps, which could indicate a problem with the BMS sending commands. The increasing deltaSupplyTemp causes the fault to become approximately 35% more likely. The cooling valve being closed contributes another 5%. Ambient temperature adds to the “cooling coil valve closed” type of fault, which could be a result of the effect that ambient temperature has when the cooling coil should be utilized.

### 4.5. Expert Survey

We conducted a survey with seven HVAC engineers, who had also previously provided input about the domain requirements to evaluate the fault diagnosis explanations and visualization techniques. In the experiment, participants were shown three different types of graphical representation for fault explanations:

(a) Standard SHAP plot for individual instances;
(b) Standard SHAP stacked plot for a specific time period;
(c) Modified version of the SHAP explanation.

The experiment was conducted as a written survey. The complete questions can be found in the Appendix A. Two types of faults were tested: “fan pressure sensor malfunction” and “heating coil valve leak”.

First, the SHAP standard plot for individual instances is shown to explain one fault sample. Then, the SHAP standard plot for explaining multiple instances is shown for a similar fault sample. Furthermore, our version of the SHAP explanation is then provided, where the relevant sets of features are shown. The participants were asked to confirm whether the explanations represented the actual fault and to rate their satisfaction with each type of visualization technique. They were also asked for their opinion on what the important criteria were to help in the fault diagnosis decision-making process.

#### Expert Insights

**Explanation assessment**: In general, the participants rated the modified SHAP explanation plots (c) the highest, and they also showed the most satisfaction with the visual representation from (c). Table 6 shows the explainability score and user satisfaction score obtained from the survey. The mean and median ratings are provided. Since users were asked to rate the explanations using a 5-point Likert scale, the explainability score was converted into values from 1 to 5 to obtain a numeric metric. For both fault types, participants typically agreed more with explanation (c) than they did with (a) and (b). For user satisfaction, explanation (c) was ranked higher than (a) and (b) on average for both of the fault cases.

**Expert Feedback**: From the numeric results obtained in the previous section, participants also had extra comments to help improve the results:

- E2 thinks that different types of faults should have their own methods of representation. Faults are different in terms of how they develop. As an example, the heating coil fault appears gradually. Therefore, an explanation for one single instance may be enough to determine the fault at the current state. The opposite case applies to faults that occur very suddenly. For example, in the case of the malfunction of the fan pressure sensor, the fault appears abruptly, and so only a short history of the fault can help identify the problem. In the case of fan pressure sensor failure, the fan pressure measurement drops so suddenly that the users are able to see the obvious change in the pattern, where the fan speed becomes very low. In comparison, the faults that develop gradually may not always be obvious from only a short history.
- E1 would like further improvement in the visualization. The participant also mentions that the plot contains a lot of visual noise. This is not a problem with measurement values that are more static. However, it may become a problem if there are too many values that change over the short visualization history. Currently, all of the annotation labels for each variable are highlighted with the same color, either green or red. Therefore, more colors would help to distinguish between different variables and make it easier to follow.
- E5 gives positive feedback, but would also like to see more related variables that would help validate the impact of the fault. The cost impact, thermal comfort impact, and component lifespan impact would help in understanding the importance of the fault and allow the HVAC engineers to prioritize maintenance activities accordingly.
- E4 commented on the fault type “fan pressure sensor malfunction” for which the features shown are relevant. However, the participants pointed out that the supply fan pressure should receive more weight than the supply fan speed itself, the opposite of which is given in the explanation graph. However, this can take the form of either the auto-generated feature importance from the model itself or the representation of the samples in the dataset. This is important, as users can immediately determine whether the system can be trusted. If the model gives the correct weight to the correct features, then it will build trust.
- E6 commented on the “heating coil valve leakage fault” to say that the explanations given are sufficient, but there are further uncertainties. It is difficult to say with confidence that it is a fault in the heating coil, since there can also be design problems that cause the temperature increase. It may be that the temperature measurement locations are not taken exactly before and after the coil fails; therefore, the temperature increase may instead be due to heat transfer through the long ducts. For the fault type “fan pressure malfunction”, E6 was also uncertain after looking at the short history and would also like to view the history from the previous day in order to understand whether the fault was caused by the building’s schedule.

**Analysis of the Explanation**: For both types of fault in the questionnaire, when presented with plot (c) and asked to assess the explainability, most participants picked the answer “Strongly Agree”, while they picked “Neutral” or “Somewhat Agree” for plots (a) and (b). After further analysis of the participants’ feedback from the survey, we summarize the findings as follows:

1. Users would like to visualize relevant features. This includes the most important features impacting the fault and the features that would further aid in confirming or rejecting the fault.
2. Users want to see how frequently the fault has appeared. Therefore, more flexibility in the sliding window graph is required, i.e., an option to view the history from the day before the fault occurs, or even earlier.
3. Users want to have a reference point, i.e., expected values, which they can compare with the values of the faulty sample.

## 5. Discussion

In this paper, we presented an approach for explaining some common air-handling unit faults identified by an XGBoost Classifier using SHAP. We demonstrate the potential of SHAP as an explainable method to aid in the user decision-making process in the field of building energy systems. We adopted XAI into the data-driven fault detection and diagnosis pipeline and explored different ways to communicate the faults to end-users, who were HVAC engineers. We defined relevant sets of features for each type of fault using domain knowledge. Then, we integrated pre-defined features into the explanation visualization and removed features with low SHAP values to reduce the visual noise. From a survey with domain experts, we learnt that some faults require an understanding of what has happened at earlier time steps in order to identify the problems. In this regard, fault diagnosis was explained based on sliding window observations. Then, we evaluated the influence of the generated explanation on the users’ decision-making process as compared to the standard SHAP explanation plots. The survey findings indicate that, on average, the confidence that users have in the fault diagnosis improved with the generated explanations, as did user satisfaction. In our work, we integrated domain knowledge to guide the design of the fault detection and diagnosis pipeline and to analyze the feature attributions of the model for each type of fault. This provided us with further insights into how the fault diagnosis information is perceived by HVAC engineers.

### Limitations

This paper presents an approach for communicating fault explanations to end-users in the HVAC domain to help build trust in the decision-making process. However, this study has limitations, as follows:

1. The current work is limited to a dataset from a single air-handling unit. It may be possible to use the model to diagnose the specific AHU used to train the model. However, aggregating datasets from multiple AHUs would be useful in generalizing the faults, and would also help in studying the practicality of the data-driven method for large-scale fault diagnosis systems. Then, its usage will not be restrained to only one machine.
2. Related to the previous point, our dataset contains only the minimum number of variables. Because of sensor costs, building owners tend to install the minimum number of sensors necessary for control. Therefore, not all of the sensors required for fault detection tasks are available. Features such as meter values that may provide valuable information on the “heating coil valve leakage” fault are not always measured. Although the faults can be identified based on the fault symptoms that appear, it is still difficult to pinpoint with full certainty what the causes of the fault are in the case that a crucial sensor is missing. Thus, the explanation capability is limited, and users are not convinced of the predicted fault after being provided with the explanation.
3. This work is only limited to one type of sensor fault, which is available in the dataset. Sensor faults are very common for building systems. Examples may include sensor bias and sensor drift. This problem may cause uncertainties in fault diagnosis since the system may read the wrong measurement and classify the samples as anomalies. Therefore, identifying sensor faults would mitigate the risk of incorrect diagnoses and false alarms. Further analysis of sensor faults is crucial for fault diagnosis tasks.
4. In this work, the definition of a good explanation is limited to only the scores from user evaluation. However, a more systematic approach would produce a more accurate evaluation and make the tasks less manual. Human users may make mistakes in the evaluation process, and one user may also perceive the fault explanations differently from another user.
5. The AHU samples used in this study were collected during the COVID-19 period. During this period, the ventilation units of the shopping mall experienced unusual loads, due to changing government regulations that impacted building occupancy rates. Therefore, the faults may have appeared more frequently and may not reflect the typical conditions of the AHU of the building.

## 6. Conclusions and Future Work

Advanced machine learning techniques have recently demonstrated excellent performance in fault detection and diagnosis problems. Nevertheless, building personnel may find it hard to evaluate and understand the reasoning behind the produced outputs. In this light, we developed an approach that utilizes the game-theory-based SHAP method to explain the output of an XGBoost classifier for fault detection and diagnosis tasks. We tackled the challenge of communicating the fault diagnosis information to HVAC engineers using the feature selection technique and sliding window analysis. The survey results from seven HVAC engineers show that with our method, the visual perception and fault diagnosis confidence were improved compared to the default explanations generated by SHAP. The study offers a general-purpose framework applicable to fault detection and diagnosis. It can be generalized to different scenarios in a broad range of areas, such as the building domain and the energy system domain. The presented work, demonstrated using real commercial building data, can give us more confidence to adopt explainable methods in real-life applications. As we only adopted one XAI method in the current study, in future work, we may involve a domain expert to evaluate and compare the explanations generated by multiple different XAI methods, such as LIME, and confirm their contextual importance and utility by using different HVAC datasets.

### Future Work

From the research findings and limitations addressed in the previous sections, the following research directions are suggested for future study:

1. The explanation and visualization methods should be further improved to cover more types of faults in a more extended dataset. For example, different types of faults, such as gradual faults and abrupt faults, may require different methods in order to be explained. For some faults, a longer history should be visualized, while for others, an explanation from only an individual instance is sufficient. More extensive involvement from HVAC engineers is necessary in order to provide explanations that suit various types of faults and that are effective in communicating information to end-users.
2. We aim to strengthen the definition of a good explanation for fault detection in the HVAC systems of buildings. In addition to user evaluation, we should adopt a further systematic approach that is less reliant on manual evaluation.
3. A larger dataset is required in order to test the scalability of the fault diagnosis method. The dataset may include samples from the AHU of different buildings and under different climate conditions. Other HVAC components, such as chillers or heat pumps, may also be explored. Ideally, more types of faults will be covered, including sensor faults and component faults.
4. Other challenging possibilities also include the development of an unsupervised model for fault detection tasks, and the application of an explainable method to understand the output.

## Figures and Tables

**Table 1:** The summary of FDD methods, fault classes, and the numerical results of the best-performing methods for each study for air-handling units in the literature.

Ref.	Application	AI Model	XAI Technique	Number of Fault Classes	Evaluation Metrics	Fault Classes	F1-Score	Year
This work	Detecting AHU faults	XGBoost, RF, Logistic Regression	SHAP	5	Accuracy, precision, recall, sensitivity, specificity, F1-score	Heating coil valve leak	0.978	2022
						Heat recovery fault	0.997	
						Cooling coil valve stuck	0.986	
						Fan pressure sensor failure	0.998	
						Cooling coil valve closed	1.00	
[18]	Detecting AHU faults	Rule and 1D-CNN	N/A	4	F1-score	Cooling coil valve stuck	0.987	2021
						Fan circuits broke down	0.995	
						Outdoor air excessive	0.993	
						Unit air leakage	0.985	
[24]	Detecting AHU faults	Multiscale convolutional neural networks	N/A	4	F1-score	Duct air leakage	1.00	2021
						Fan efficiency decrease	0.999	
						Cooling coil valve stuck	0.994	
						Outdoor air excess	1.00	
[25]	Detecting heat recycler faults in the AHU	SVM and NN	LIME	1	Accuracy, recall, precision, sensitivity, specificity, F1-score	Heat recycler failure	1.00	2019
[26]	Detecting AHU faults	Decision tree	N/A	8	F1-score	Heating coil valve leakage	0.90	2016
						Cooling coil valve stuck	1.00	
						Cooling coil valve stuck at 65%	0.98	
						Return fan fixed at 30%	1.00	
						Return fan failure	1.00	
						Outdoor air damper stuck	1.00	
						Exhaust air damper stuck	0.90	
						Duct leakage before supply fan	1.0	

**Table 2:** Description of the used features in the AHU dataset.

No.	Feature	Short Description	Unit
x1	AAT	Fresh air intake temperature	°C
x2	ACCVO	Cooling coil valve opening	%
x3	AHCVO	Heating coil valve opening	%
x4	AHRS	Heat recovery rotation speed	%
x5	AHRST	Supply air temperature after heat recovery	°C
x6	ARAT	Return air temperature	°C
x7	ASAT	Supply air temperature	°C
x8	ASATCSP	Supply air temperature calculated setpoint	°C
x9	ASFPE	Supply fan static pressure	Pa
x10	ASFS	Supply fan speed	%
x11	tempDiffHC	Temperature difference before and after heating coil	°C
x12	HREfficiency	Heat recovery efficiency	%
x13	deltaSupplyTemp	Difference between supply air temperature and supply air temperature setpoint	°C

**Table 3:** List of AHU faults used in the analysis.

No.	Abbreviation	Fault Type	Component	Sample Size
Fault 1	FPES_M	Fan pressure sensor malfunction	Fan pressure sensor	894
Fault 2	HR_NW	Heat recovery not working	Heat recovery	1146
Fault 3	HCV_L	Heating coil valve leakage	Heating coil	794
Fault 4	CCV_S	Cooling coil valve stuck	Cooling valve	434
Fault 5	CCC_V	Closed cooling coil valve	Control	768
–	Normal	–	–	20,925

**Table 4:** Mapping features to their corresponding faults.

	x1	x2	x3	x4	x5	x6	x7	x8	x9	x10	x11	x12	x13
Fault 1									✓	✓			
Fault 2	✓			✓		✓	✓					✓	
Fault 3			✓		✓		✓				✓		
Fault 4		✓					✓	✓					✓
Fault 5		✓					✓	✓					✓

✓ indicates that the feature is used for predicting the fault.

**Table 5:** Performance matrix of the used models in the fault diagnosis task.

Model	Fault Class	Accuracy	Precision	Recall	Sensitivity	Specificity	F1
**LR**	HCV_L	0.981	0.731	0.828	0.828	0.987	0.776
	HR_NW	0.995	0.932	0.971	0.971	0.996	0.951
	CCV_S	0.997	0.888	0.972	0.972	0.997	0.928
	FPES_M	0.999	0.996	1	1	0.999	0.998
	CCV_C	0.985	0.791	0.748	0.748	0.993	0.769
	Normal	0.883	0.954	0.889	0.889	0.861	0.920
	**Weighted**	0.859	0.943	0.900	0.900	0.893	0.887
**RF**	HCV_L	0.997	0.955	0.988	0.988	0.988	0.971
	HR_NW	0.999	0.997	0.997	0.997	0.999	0.997
	CCV_S	0.999	0.960	0.986	0.986	0.999	0.973
	FPES_M	0.999	0.996	1	1	0.999	0.998
	CCV_C	0.999	0.999	0.989	0.989	0.999	0.993
	Normal	0.995	0.996	0.994	0.994	0.998	0.997
	**Weighted**	0.993	0.996	0.994	0.994	0.998	0.994
**XGB**	HCV_L	0.998	0.974	0.982	0.982	0.998	0.978
	HR_NW	0.999	0.997	0.997	0.997	0.999	0.997
	CCV_S	0.999	0.973	1	1	0.999	0.986
	FPES_M	0.999	0.996	1	1	0.999	0.998
	CCV_C	1	1	1	1	1	1
	Normal	0.997	0.998	0.997	0.997	0.996	0.998
	**Weighted**	0.996	0.997	0.997	0.997	0.997	0.997

LR—logistic regression; RF—random forest; XGB—XGBoost.

**Table 6:** Mean and median values of explainability score and user satisfaction for the fault types “fan pressure sensor not working” (FPES_M) and “heating coil valve leakage” (HCV_L) using three different types of visualization.

		FPES_M			HCV_L		
	Measures	(a)	(b)	(c)	(a)	(b)	(c)
Explainability score	mean	3	2.71	3.71	3.14	2.28	4.71
	median	4	2	5	4	1	5
User satisfaction	mean	5.71	6.57	9	5.71	5.85	7.85
	median	6	7	9	6	6	9

**Figure A1:**
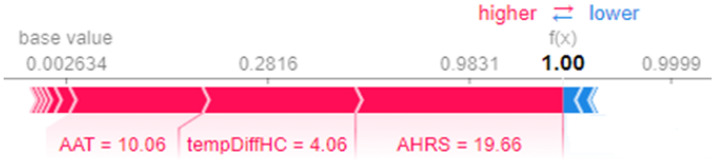
Heating coil valve leakage. Standard SHAP plot for individual explanations.

**Figure A2:**
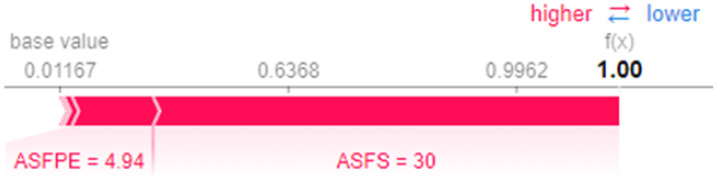
Fan pressure sensor malfunction. SHAP explanation for fault at a specific timestamp.

**Figure A3:**
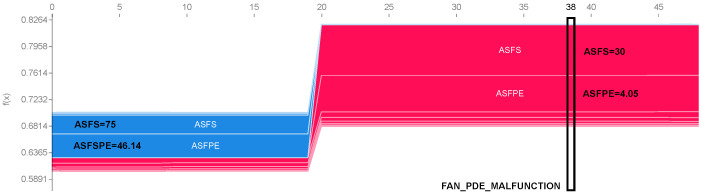
Fan pressure sensor malfunction. SHAP plot for sliding window explanation visualized from “03-11 10:00” to “03-11 22:00”; *x* axis represents 15 min time instances.

**Figure A4:**
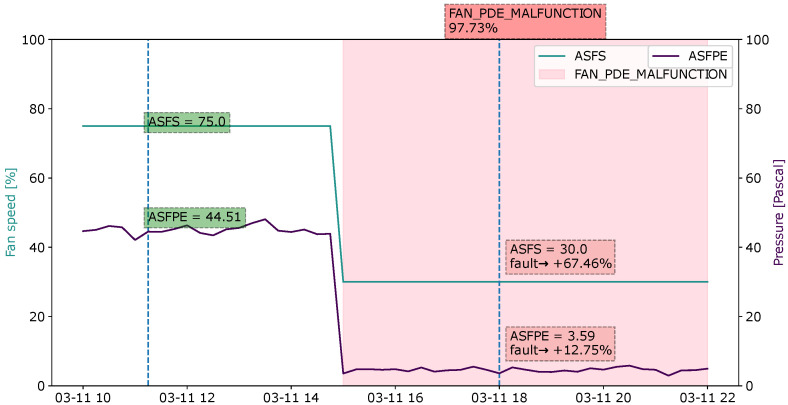
Fan pressure sensor malfunction. Modified SHAP plot for sliding window explanation; *x* axis represents 15 min time instances.

**Figure A5:**
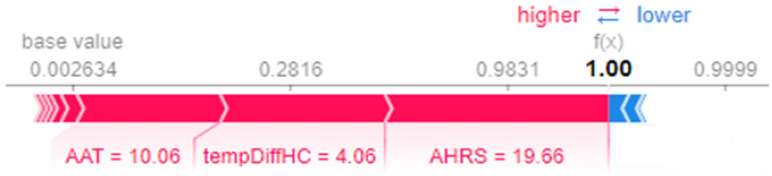
Heating coil valve leakage. Standard SHAP plot for individual explanations.

**Figure A6:**
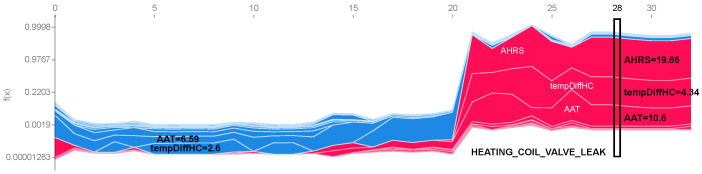
Heating coil valve leakage. SHAP plot for sliding window explanation, visualized from “03-25 08:00” to “03-25 16:00”.

**Figure A7:**
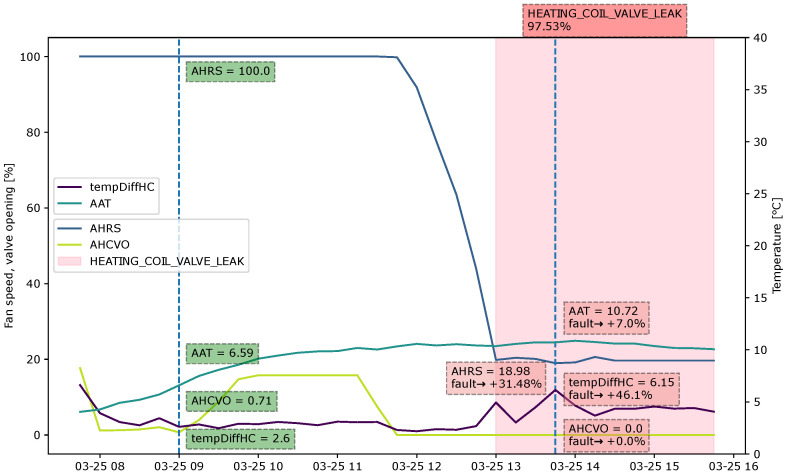
Heating coil valve leakage. Modified SHAP plot for sliding window explanation.

## Data Availability

Not applicable.

## Abbreviations

The following abbreviations are used in this manuscript:

1D-CNN	One-Dimensional CNN
AHU	Air-Handling Unit
AI	Artificial Intelligence
API	Application Programming Interface
BMS	Building Management System
CNN	Convolutional Neural Network
FDD	Fault Detection and Diagnosis
Grad-Absolute-CAM	Absolute Gradient-weighted Class Activation Mapping
HVAC	Heating, Ventilation and Air Conditioning
LIME	Local Interpretable Model-Agnostic Explanations
ML	Machine Learning
NN	Neural Network
SHAP	Shapley Additive Explanations
SVM	Support Vector Machine
XAI	Explainable Artificial Intelligence
XGBoost	Extreme Gradient Boosting

## Appendix A. Explainable Fault Detection and Diagnosis Methods for Air-Handling Units: Research Survey

**Goal:** The goal of the survey was to assess the explanations’ influence on the decision making for fault diagnosis tasks and to compare various visualization techniques. The focus is on the delivery of the explanations.

**Domain Requirements**

This is a sample of an auto-generated explanation for AHU faults, where the variables highlighted in red and blue are variables that have a positive and negative influence on the fault likelihood, respectively.

Time: “03-25 15:45”

Predicted fault: Heating coil valve leak

Fault probability: 1.00

Based on the above plot, please **rate**, on a scale of 1 to 10, how important these criteria are in helping to understand whether a fault explanation actually represents a fault.

**CR1:** I want to be able to choose and view more variables (i.e., AHCVO) in addition to the auto generated features as shown in the plot above (AAT, tempDiffHC, AHRS). ______ /10

**CR2:** I want to be able to visualize the short history of fault to understand what has happened prior to the fault. ______ /10

**CR3:** I want to be able to view only the most important variables that influence the likelihood of the fault. ______ /10

**CR4:** I want to view in terms of probability how much each feature affects the fault likelihood. ______ /10

Other important criteria you would like to add: ______________________________

**Visualization Design**

The following are the sample explanations for AHU faults visualized in three different ways to explain each type of fault. Please indicate if the explanation is convincing and rate on the scale of 1 to 10 your satisfaction with each type of visualization.

* All the samples shown here are observations only when AHU is switched on.

**Predicted Fault: Fan Pressure Sensor Malfunction**

(a).

Time: “03-11 16:00”

Fault probability: 1.00

Does the above plot explain fan pressure sensor malfunction? (Strongly Agree/Somewhat Agree/Neutral/Somewhat Disagree/Strongly Disagree) ______

Your satisfaction with the graphical representation: ______ /10

(b).

Time: “03-11 19:45”

Fault probability: 0.99

Does the above plot explain fan pressure sensor malfunction? (Strongly Agree/Somewhat Agree/Neutral/Somewhat Disagree/Strongly Disagree) ______

Your satisfaction with the graphical representation: ______ /10

(c).

Time: “03-11 18:00”

Fault probability: 97.73%

Does the above plot explain fan pressure sensor malfunction? (Strongly Agree/Somewhat Agree/Neutral/Somewhat Disagree/Strongly Disagree) ______

Your satisfaction with the graphical representation: ______ /10

**Predicted Fault: Heating coil valve leakage**

(a).

Time: “03-25 15:45:00”

Fault probability: 1.00

Does the above plot explain fan pressure sensor malfunction? (Strongly Agree/Somewhat Agree/Neutral/Somewhat Disagree/Strongly Disagree) ______

Your satisfaction with the graphical representation: ______ /10

(b).

Time: “03-25 15:00”

Fault probability: 0.99

Does the above plot explain fan pressure sensor malfunction? (Strongly Agree/Somewhat Agree/Neutral/Somewhat Disagree/Strongly Disagree) ______

Your satisfaction with the graphical representation: ______ /10

(c).

Time: “03-25 13:45”

Fault probability: 97.53%

Does the above plot explain fan pressure sensor malfunction? (Strongly Agree/Somewhat Agree/Neutral/Somewhat Disagree/Strongly Disagree) ______

Your satisfaction with the graphical representation: ______ /10
